# Analysis for bioconvection due to magnetic induction of Casson nanoparticles subject to variable thermal conductivity

**DOI:** 10.1038/s41598-024-59478-6

**Published:** 2024-04-29

**Authors:** D. K. Almutairi

**Affiliations:** Department of Mathematics, College of Science Al-Zulfi, Majmaah University, 11952 Al-Majmaah, Saudi Arabia

**Keywords:** Casson fluid, Iinduced magnetic force, Nanofluid, Microorganisms, Variable thermal conductivity, Numerical simulations, Energy science and technology, Engineering, Mathematics and computing

## Abstract

Owing to valuable significance of bioconvective transport phenomenon in interaction of nanoparticles, different applications are suggested in field of bio-technology, bio-fuels, fertilizers and soil sciences. It is well emphasized fact that thermal outcomes of nanofluids can be boosted under the consideration of various thermal sources. The aim of current research is to test the induction of induced magnetic force in bioconvective transport of non-Newtonian nanofluid. The rheological impact of non-Newtonian materials is observed by using Casson fluid with suspension of microorganisms. The chemical reaction effected are interpreted. The thermal conductivity of material is assumed to be fluctuated with temperature fluctuation. The flow pattern is endorsed by stretching surface following the stagnation point flow. Under the defined flow assumptions, the problem is formulated. A computational software with shooting technique is used to present the simulations. A comprehensive analysis for problem is presented. It is claimed that the interpretation of induced magnetic force exclusively enhanced the thermal phenomenon.

## Introduction

In the modern world, the continuous research in nanotechnogly brought out the idea of nanofluid which is widely studied due to efficient thermal outcomes. Being tiny structure and micro-sized, nanoparticles attain exclusive thermal predictions and high heating performances. Various applications characterize the significance of such particles due to boosted thermal features. Some common applications of nanomaterials are observed in heat emission processes, nano-biosensors, heat transfer deices, cleaning the surfaces, charge transport, solar cells, semiconductors, chemical processes etc. Choi^1^ provided primary attention of nanoparticles in an experimental work and proved that base fluid properties can be boosted upon interaction of metallic particles. Rashid et al.^2^ studied the titanium and silver thermal combination while exploring the improvement in heat transfer. Jalili et al.^3^ used the python computational software to present the computations for nanofluid problem in vertical channel. The micropolar nanofluid attention in porous media via comprehensive review was endorsed by Pop et al.^4^. Elmonem et al.^5^ announced the rotating disk flow with MoS2 nanoparticles with heat generation impact. Thumma et al.^6^ discussed the Hall features for nanofluid and reported optimizing results for heat transfer phenomenon. Kolsi et al.^7^ intended bidirectional flow in oscillating regime under the assumptions of nanoparticles having diverse viscosity. The mixed convection onset regarding the hybrid nanofluid against cone flow was intended by Paul et al.^8^. Obalalu et al.^9^ explored the entropy generation assessment of nanofluid with solar power device. Yang et al.^10^ executed the blade and platelet consequences for nanofluid with slip flow. Yasir et al.^11^ investigated the axisymmetric flow due to hybrid nanofluid with utilization of heat source. Wan et al.^12^ focused on stable properties of silicone-oil nanofluid at high temperature. A double diffusion flow of nanofluid in inclined surface was predicted by Batool et al.^13^. Nazir et al.^14^ explained the thermal change in properties of ethylene glycol with interaction of titania nanoparticles. Sohail et al.^15^ focused to the investigation of boosted heat phenomenon by using the two distinct hybrid nanoparticles by using the Galerkin finite element technique. Elboughdiri et al.^16^ explored the solar applications with significant thermal outcomes due to nanofluid accounted by cone. The Dufour impact in Prandtl number fluid flow via vertically moving surface was reported by Liu et al.^17^. Waqas et al.^18^ endorsed the nanofluid properties through porous channel with radiated impact. Waqas et al.^19^ explored the gold nanoparticles thermal measurements due to stenotic artery flow. In another approach Waqas et al.^20^ analyzed the comparative analysis for heat transfer due to hybrid nanofluid. The prediction of enhanced thermal features due to nanofluid with optimized aspects was examined by Waqas et al.^21^.

The induced magnetic force is an interesting phenomenon associated to the electromagnetic induction. The fluctuation in the magnetic force in close surface endorsed an electromotive force based on theory of Faraday’s law. Various applications of electromagnetic force are observed in engineering science, plasma physics and technological processes. The most valuable application of this phenomenon is the magnetic resonance mage (MRI). The MRI is associated to the induction of boosted magnetic and radio waves to pronounce an image of structure of human body. The fundamental concept for MRI is the induction of magnetic field in the body tissues and predicting the signal production under the field is disrupted. Different studies are available on this topic. Mehmood et al.^22^ presented the utilization of nanofluid referred to the stagnation point phenomenon. Wubshet^23^ discussed the induction of magnetic force in Maxwell fluid via convective thermal approach. Xu et al.^24^ depicted the heat transmission in nanofluid under the interaction of induced magnetic force. Chen et al.^25^ discussed the double diffusion phenomenon supported with induced magnetic force impact. Akram et al.^26^ analyzed the radiative aspect of magnetized nanomaterials under to supposition of induced magnetic field. The Couette flow due to magnetic induction of Jeffrey fluid was proceeded by Jumanne et al.^27^.

The bioconvection is another topic of interest which is referred to the convection of various liquids preserving the microscopic level. The bioconvection impact is due to fluctuated densities due to swimming of various types of microorganisms. Commonly, in the upper regime of liquid surface, the movement of microorganisms have been observed. Such interaction of microorganisms makes the upper regime denser. An instability phenomenon in the flow system is noted due to such fluctuation in the densities. The bioconvection phenomenon is important in coating, enzymes, petroleum recovery, biofuels, micro-system etc. The bioconvection associated to the nanoparticles is also interesting which enhance the stability of nanomaterials. Some interesting studies on this topic are presented in refs.^28–33^.

The evaluation of above claimed research survey, it is noticed that various contributions are available for nanomaterials with distinct thermal sources. However, less attention is paid to the applications of induced magnetic force associated to the nanofluid fluid. Keeping these motivations in mind, the aim of current model is to analyze the induction of induced magnetic force for stagnation point flow of Casson nanofluid with suspension of microorganisms. The inspirations for using the Casson fluid model are due to distinct rheology. Exclusive applications of Casson fluid have been attributed in the human blood, metallurgical process, material sciences, mining industry, cosmetic etc. The flow is causing by a linearly moving stretched surface. The novel aspects of work are summered as:

- The stagnation point flow of Casson nanofluid with decomposition of microorganisms is studied.
- The induction of magnetic force is entertained for bioconvective flow of Casson nanofluid.
- The chemical reaction effects are utilized.
- The nature of variable thermal conductivity is assumed for current flow problem.
- The numerical evaluation of problem is proposed by implementing the shooting technique.
- Physical insight of problem has been examined and presented in graphically.

It is further benchmark that no recent attempt is contributed in the literature for applications of induced magnetic field with these thermal sources. The motivations for studying the applications of induced magnetic force on Casson nanofluid are due its applications in the plasma physics and high energy physics.

## Problem statement

**Figure 1:**
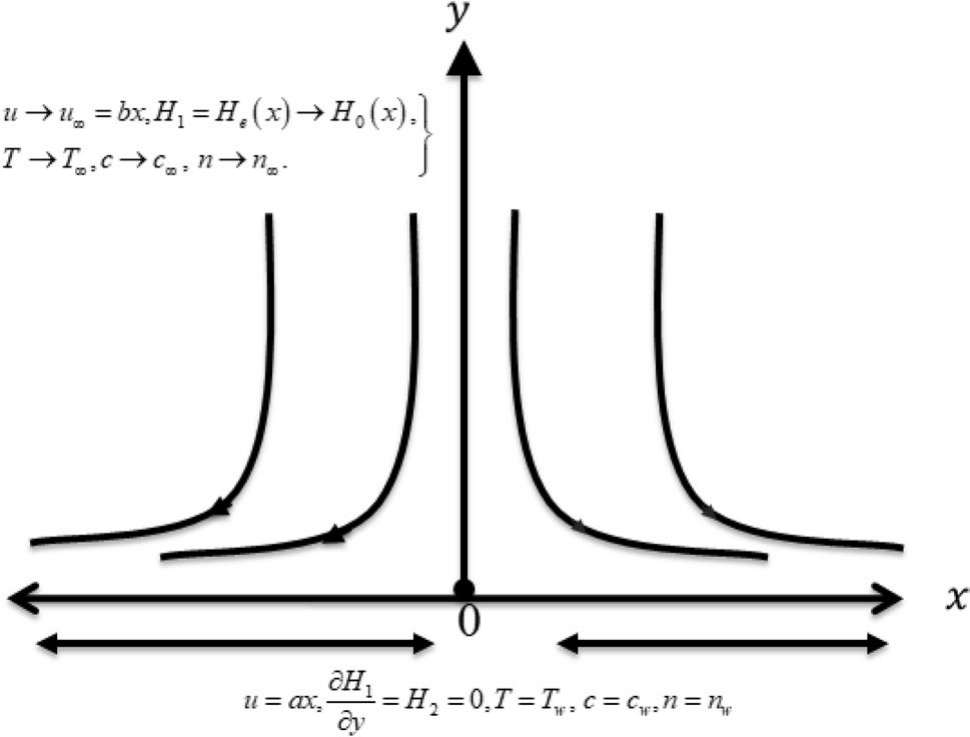
Physical visualization of the problem.

A steady flow due to Casson nanofluid with decomposition of microorganisms is detected. The flow phenomenon is accounted to the stretching surface which induced the flow with uniform velocity. The induction of magnetic force is utilized for current flow phenomenon. The cartesian plane is adopted for model the problem. The velocity attaining in free stream regime is expressed with $u = bx$ while $u = ax$ is the surface velocity with constant $a$ and $b$. For magnetic induction, let $H_{2}$ be normal component while horizontal component is expressed with $H_{1}$. It is emphasized that $H_{2}$ omitted ear the plate regime while $H_{1}$ asymptomatically approaches to $H_{0} .$ The variable thermal conductivity assumptions are taken for inspection of heat transfer impact. The normal and horizontal velocity components are denoted with $v$ and $u$, respectively. Let temperature, fluid concentration and microorganisms density is expressed via $T,c$ and $n$ respectively. The flow problem with thermal constraints is reflected in Fig. 1.

The problem is modeled in view of following governing equations:

1 $$\nabla .{\mathbf{V}} = 0,$$

2 $$\nabla .{\mathbf{H}} = 0,$$

3 $$\rho_{f} \left( {{\mathbf{V}}{\mathbf{.}}\nabla } \right){\mathbf{V}} = - \nabla p + \frac{\mu }{4\pi }\left( {\nabla .{\mathbf{H}}} \right){\mathbf{H}} + \mu \nabla^{2} {\mathbf{V}} + {\mathbf{J \times B}},$$

4 $$\nabla \times \left( {{\mathbf{V}} \times {\mathbf{H}}} \right) + \mu_{e} \nabla^{2} {\mathbf{H}} = 0,$$

5 $${\mathbf{V}}{\mathbf{.}}\nabla T = \alpha \nabla^{2} T + \tau \left[ {D_{B} \nabla T.\nabla C + \left( {\frac{{D_{T} }}{{T_{\infty } }}} \right)\nabla T.\nabla T} \right],$$

6 $$\left( {{\mathbf{V}}{\mathbf{.}}\nabla } \right)C = D_{B} \nabla^{2} C + \left( {\frac{{D_{T} }}{{T_{\infty } }}} \right)\nabla^{2} T,$$

7 $${\mathbf{V}}{\mathbf{.J}}_{{\mathbf{1}}} = 0,$$

with ${\mathbf{V}}$ (velocity vector), ${\mathbf{H}}$ (magnetic field vector),$\mu$ (dynamic viscosity), $\tau_{f}$ (ratio amongst thermal capacity of nanoparticles to fluid), $T$ (temperature), $D_{B}$ (Brownian coefficient), $D_{T}$ (thermophoresis coefficient), $C$ (concentration) and ${\mathbf{J}}_{{\mathbf{1}}}$(microorganisms flux).

In view of above governing equations, the boundary layer equations for current model are expressed as^16,17^:

8 $$\frac{\partial u}{{\partial x}} + \frac{\partial v}{{\partial y}} = 0,$$

9 $$\frac{{\partial H_{1} }}{\partial x} + \frac{{\partial H_{2} }}{\partial y} = 0,$$

10 $$u\frac{\partial u}{{\partial x}} + v\frac{\partial u}{{\partial y}} = \nu \left( {1 + \frac{1}{\beta }} \right)\frac{{\partial^{2} u}}{{\partial y^{2} }} + u_{\infty } \frac{{\partial u_{\infty } }}{\partial x} + \frac{{\sigma B_{0}^{2} }}{{\rho_{f} }}\left( {u_{\infty } - u} \right) + \mu_{e} \frac{{dU_{e} }}{dx} - \frac{\mu }{{4\pi \rho_{f} }}H_{e} \frac{{\partial H_{e} }}{\partial x} + \frac{\mu }{{4\pi \rho_{f} }}\left( {H_{1} \frac{{\partial H_{1} }}{\partial x} + H_{2} \frac{{\partial H_{1} }}{\partial y}} \right),$$

11 $$u\frac{{\partial H_{1} }}{\partial x} + v\frac{{\partial H_{2} }}{\partial y} = H_{1} \frac{\partial u}{{\partial x}} + H_{2} \frac{\partial u}{{\partial y}} + u_{e} \frac{{\partial^{2} H_{1} }}{{\partial x^{2} }},$$

12 $$u\frac{\partial T}{{\partial x}} + v\frac{\partial T}{{\partial y}} = \frac{1}{{\left( {\rho c} \right)_{f} }}\frac{\partial }{\partial y}K\left( T \right)\left( {\frac{\partial T}{{\partial y}}} \right) + \tau_{f} \left( {D_{B} \left( {\frac{\partial c}{{\partial y}}} \right)\frac{\partial T}{{\partial y}} + \frac{{D_{T} }}{{T_{\infty } }}\left( {\frac{\partial T}{{\partial y}}} \right)^{2} } \right),$$

13 $$u\frac{\partial c}{{\partial x}} + v\frac{\partial c}{{\partial y}} = \frac{{D_{T} }}{{T_{\infty } }}\frac{{\partial^{2} T}}{{\partial y^{2} }} + D_{B} \frac{{\partial^{2} c}}{{\partial y^{2} }} - k^{ * } \left( {c - c_{\infty } } \right),$$

14 $$u\frac{\partial n}{{\partial x}} + v\frac{\partial n}{{\partial y}} + \frac{{b_{m} w_{m} }}{{\left( {c_{w} - c_{\infty } } \right)}}\frac{\partial }{\partial y}\left( {n\frac{\partial c}{{\partial y}}} \right) = D_{m} \frac{{\partial^{2} n}}{{\partial y^{2} }},$$

The important physical quantities in above model are $\nu$ (kinematic viscosity), $\beta$ (Casson fluid parameter), $\rho_{f}$ (density), $\sigma$ (electrical conductivity), $u_{\infty }$ (free stream velocity), $\mu_{e}$ (magnetic diffusivity), $H_{e}$ ($x -$ magnetic field at surface), $K\left( T \right)$ (thermal conductivity as a function of temperature), $k^{ * }$ (chemical reaction coefficient), $b_{m}$ (chemotaxis constant), $w_{m}$ (maximum cell swimming) and $D_{m}$ (density of microorganisms).

For variable thermal impact, the relation for thermal conductivity is defined as:

15 $$K\left( T \right) = K_{\infty } \left( {1 + \alpha \frac{{T - T_{\infty } }}{\Delta T}} \right),$$

with thermal conductivity coefficient $\alpha$.

The problem is entertained by following constraints:

16 $$u = ax,v = 0,\frac{{\partial H_{1} }}{\partial y} = H_{2} = 0,T = T_{w} ,\,\,c = c_{w} ,n = n_{w} \,\,\,\,\,at\,\,\,\,\,y = 0,$$

17 $$u \to u_{\infty } = bx,v = 0,\,\,\,H_{1} = H_{e} \left( x \right) \to H_{0} \left( x \right),T \to T_{\infty } ,\,c \to c_{\infty } ,\,\,n \to n_{\infty } \,\,at\,\,y \to \infty .$$

The new proposed variables are:

18 $$\left. \begin{gathered} v = - \sqrt {a\nu } f\left( \eta \right),H_{2} = - H_{0} \sqrt {\frac{\nu }{a}} g\left( \eta \right),\eta = \sqrt {\frac{a}{\nu }} y,u = axf^{\prime}\left( \eta \right), \hfill \\ \phi \left( \eta \right) = \frac{{c - c_{\infty } }}{{c_{w} - c_{\infty } }},\theta \left( \eta \right) = \frac{{T - T_{\infty } }}{{T_{w} - T_{\infty } }},\chi \left( \eta \right) = \frac{{n - n_{\infty } }}{{n_{w} - n_{\infty } }}. \hfill \\ \end{gathered} \right\}.$$

19 $$\left( {1 + \frac{1}{\beta }} \right)f^{\prime \prime \prime } - f^{\prime 2} + ff^{\prime \prime } + C^{2} + \lambda \left( {g^{\prime 2} - gg^{\prime \prime 2} - 1} \right) = 0,$$

20 $$\omega g^{\prime\prime\prime} + fg^{\prime\prime} - gf^{\prime\prime} = 0,$$

21 $$\left( {1 + \alpha \theta } \right)\theta^{\prime\prime} + \Omega \left( {\theta^{\prime}} \right)^{2} \theta^{\prime\prime} + Pr\left[ {Nb\theta^{\prime}\phi^{\prime} + f\theta^{\prime} + Nt\left( {\theta^{\prime}} \right)^{2} } \right] = 0,$$

22 $$\phi ^{\prime\prime} - ScKr\phi + \left( {\frac{Nt}{{Nb}}} \right)\theta^{\prime\prime} + Scf\phi^{\prime} = 0,$$

23 $$\chi ^{\prime\prime} + Lbf\chi ^{\prime} - Pe\left( {\phi ^{\prime\prime}\left( {\chi + \sigma_{m} } \right) + \chi ^{\prime}\phi ^{\prime}} \right) = 0.$$

with boundary conditions:

24 $$\left. \begin{gathered} f\left( 0 \right) = 0,f^{\prime}\left( 0 \right) = 1,g\left( 0 \right) = g^{\prime\prime}\left( 0 \right) = 0, \hfill \\ \theta \left( 0 \right) = 0,\phi \left( 0 \right) = 1,\chi \left( 0 \right) = 1, \hfill \\ \end{gathered} \right\}$$

25 $$\left. \begin{gathered} f^{\prime}\left( \infty \right) \to C,\,\,\,\,\,\,\,\,\,\,g^{\prime}\left( \infty \right) \to 1,\,\,\,\,\,\,\,\,\theta \left( \infty \right) \to 0, \hfill \\ \phi \left( \infty \right) \to 0,\,\,\,\,\,\,\,\,\,\,\,\,\,\,\chi \left( \infty \right) \to 0. \hfill \\ \end{gathered} \right\}$$

with dimensionless variables magnetic parameter $\lambda = \frac{\mu }{{4\pi \rho_{f} }}\left( {\frac{{H_{0} }}{a}} \right)^{2} ,$ velocity ratio $C = \frac{b}{a},$ reciprocal magnetic Prandtl number $\omega = \frac{{\mu_{e} }}{\nu },$ chemical reaction constant $Kr = \frac{{k^{ * } }}{a}$, Lewis number $Sc = \frac{\nu }{{D_{B} }}$, bio-convective Lewis number $Lb = \frac{\nu }{{D_{m} }},$ Brownian constant $Nb = \frac{{\tau_{f} D_{B} \left( {C_{w} - C_{\infty } } \right)}}{\nu },$ Peclet number $Pe = \frac{{b_{m} w_{m} }}{{D_{m} }}$, motile difference constant $\sigma_{m} = \frac{{n_{\infty } }}{{\left( {n_{w} - n_{\infty } } \right)}}$ and thermophoresis constant $Nt = \frac{{\tau_{f} D_{T} \left( {T_{w} - T_{\infty } } \right)}}{{T_{\infty } \nu }}$.

The Nusselt number, Sherwood number and motile density number are:

26 $$\left. \begin{gathered} \left( {{\text{Re}}_{x} } \right)^{ - 1/2} Nu = - \theta ^{\prime}\left( 0 \right), \hfill \\ \left( {{\text{Re}}_{x} } \right)^{ - 1/2} Sh = - \phi ^{\prime}\left( 0 \right), \hfill \\ \left( {{\text{Re}}_{x} } \right)^{ - 1/2} Nn = - \chi ^{\prime}\left( 0 \right). \hfill \\ \end{gathered} \right\}$$

## Computational analysis

**Figure 2:**
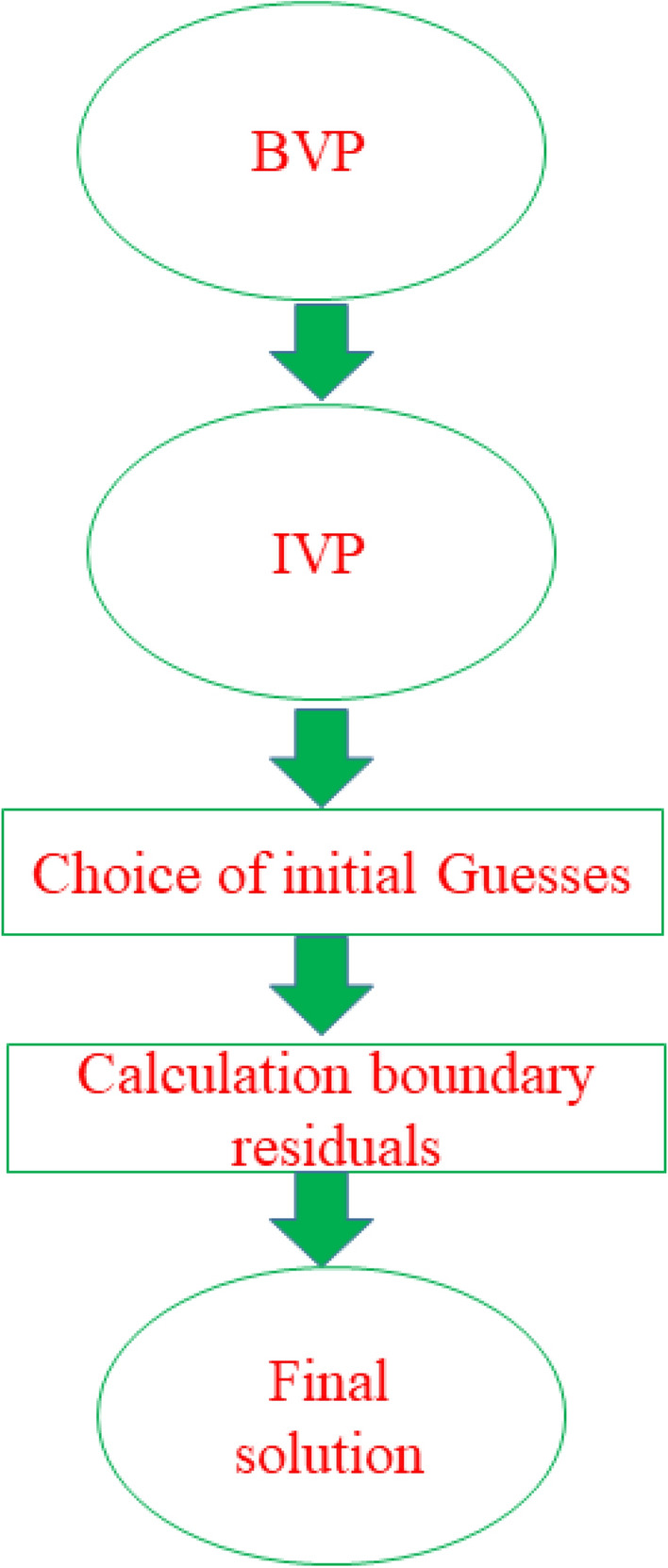
Numerical flow chart of scheme.

The problem modeled in the previous section containing highly nonlinear as well as well couple equations. Such equations are solved via computational scheme namely shooting method, according to Fig. 2. The benefit and motivations of this tool is due to high accuracy and less error. The problem is convert into first order approximations as follows:

27 $$\left. \begin{gathered} f = j_{1} , \, f^{\prime} = j_{2} , \, f^{\prime\prime} = j_{3} ,f^{\prime\prime\prime} = j_{3}{\prime} ,g = j_{4} {,}g^{\prime} = j_{5} {,}g^{\prime\prime} = j_{6} {,}g^{\prime\prime\prime} = j_{6}{\prime} {, } \hfill \\ \, \theta = j_{7} , \, \theta^{\prime} = j_{8} ,\theta^{\prime\prime} = j_{9}{\prime} , \, \hfill \\ \phi = j_{10} , \, \phi^{\prime} = j_{11} ,\phi^{\prime\prime} = j_{12}{\prime} ,\chi = j_{13} , \, \chi^{\prime} = j_{14} ,\chi^{\prime\prime} = j_{14}{\prime} , \hfill \\ \end{gathered} \right\}$$

The new developed problem is:

28 $$f^{\prime\prime\prime} = \frac{{j_{2}^{2} - j_{1} j_{3} - C^{2} - \lambda \left( {j_{5}^{2} - j_{4} j_{6} - 1} \right)}}{{1 + \frac{1}{\beta }}},$$

29 $$j^{\prime}_{6} = \frac{1}{\omega }\left( {j_{1} j_{6} - j_{4} j_{3} } \right),$$

30 $$j^{\prime}_{9} = \frac{{ - \alpha \left( {j_{8} } \right)^{2} - Pr\left[ {Nbj_{8} j_{11} + j_{1} j_{8} + Nt\left( {j_{8} } \right)^{2} } \right]}}{{1 + \Omega j_{7} }},$$

31 $$j^{\prime}_{12} = ScKrj_{10} - \left( {\frac{Nt}{{Nb}}} \right)j^{\prime}_{9} - Scj_{1} j_{11} ,$$

32 $$j_{14}{\prime} = - Lbj_{1} j_{14} + Pe\left( {j^{\prime}_{12} \left( {j_{13} + \sigma_{m} } \right) + j_{14} j_{11} } \right).$$

with boundary conditions:

33 $$\left. \begin{gathered} j_{1} \left( 0 \right) = 0,j_{2} \left( 0 \right) = 1,j_{4} \left( 0 \right) = j_{6} \left( 0 \right) = 0, \hfill \\ j_{7} \left( 0 \right) = 0,j_{10} \left( 0 \right) = 1,j_{13} \left( 0 \right) = 1. \hfill \\ \end{gathered} \right\}$$

34 $$\left. \begin{gathered} f^{\prime}\left( \infty \right) \to C,\,\,\,\,\,\,\,\,\,\,g^{\prime}\left( \infty \right) \to 1,\,\,\,\,\,\,\,\,\theta \left( \infty \right) \to 0, \hfill \\ \phi \left( \infty \right) \to 0,\,\,\,\,\,\,\,\,\,\,\,\,\,\,\chi \left( \infty \right) \to 0. \hfill \\ \end{gathered} \right\}$$

The computations are performed with excellent accuracy of 10^–8^.

## Verification of numerical results

In order to evaluates the solution validity and accuracy, the computed data is compared with investigations of Mehmood and Iqbal^22^ and Ali et al.^34^ in Table 1. The simulated results convey fine accuracy with these studies.

**Tabel 1:** Verification of numerical data with available studies when $\lambda = 0,\beta \to \infty .$

$C$	Mehmood and Iqbal^22^	Ali et al.^34^	Present results	CPU time (s)
0.1	− 0.9694	− 0.9694	− 0.96950	0.255
0.2	− 0.9181	− 0.9181	− 0.91812	0.521
0.5	− 0.6673	− 0.6673	− 0.66732	1.325

## Physical impact of problem

**Figure 3:**
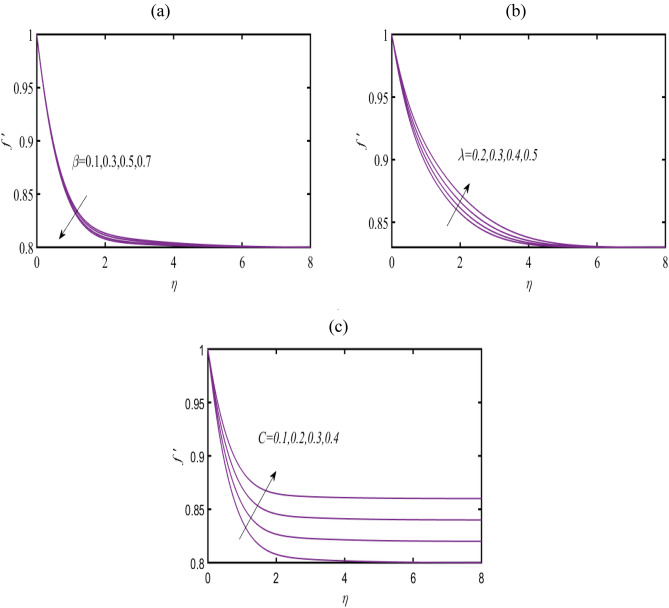
(**a**–**c**): Prediction of $f^{\prime}$ for (**a**) $\beta$ (**b**) $\lambda$ and (**c**) $C.$

The demonstration of physical phenomenon is important and analyzed in this section. The defined problem is subject to theoretical flow constraints due to which fixed numerical values have been allotted to involved parameters like $$\lambda = 0.5,$$ $$C = 0.2,$$ $$\omega = 0.4,$$ $$Kr = 0.3,$$ $$Sc = 0.5$$, $$Lb = 0.2,$$ $$Nb = 0.3,$$, $$Pe = 0.5$$$$\sigma_{m} = 0.1,$$ $$Nt = 0.3.$$ Figure 3a comprising the significance of Casson parameter $$\beta$$ on velocity profile $$f^{\prime}$$. The reduction in the profile of $$f^{\prime}$$ is deduced due to $$\beta$$. Such impacted results are associated to the distinct rheology of Casson fluid model. Physically, the viscosity of fluid become thicker against larger $$\beta$$ which declining the velocity. Figure 3b classifying the onset of magnetic constant $$\lambda$$ in prediction of $$f^{\prime}$$. Larger influence of $$\lambda$$ on velocity is disclosed. Such features are physical associated to the interaction of magnetic induction. For observing the physical sense of velocity ratio $$C$$ on $$f^{\prime}$$, Fig. 3c is prepared. The increasing trend of $$f^{\prime}$$ is predicted due to enhancement in $$C$$.

**Figure 4:**
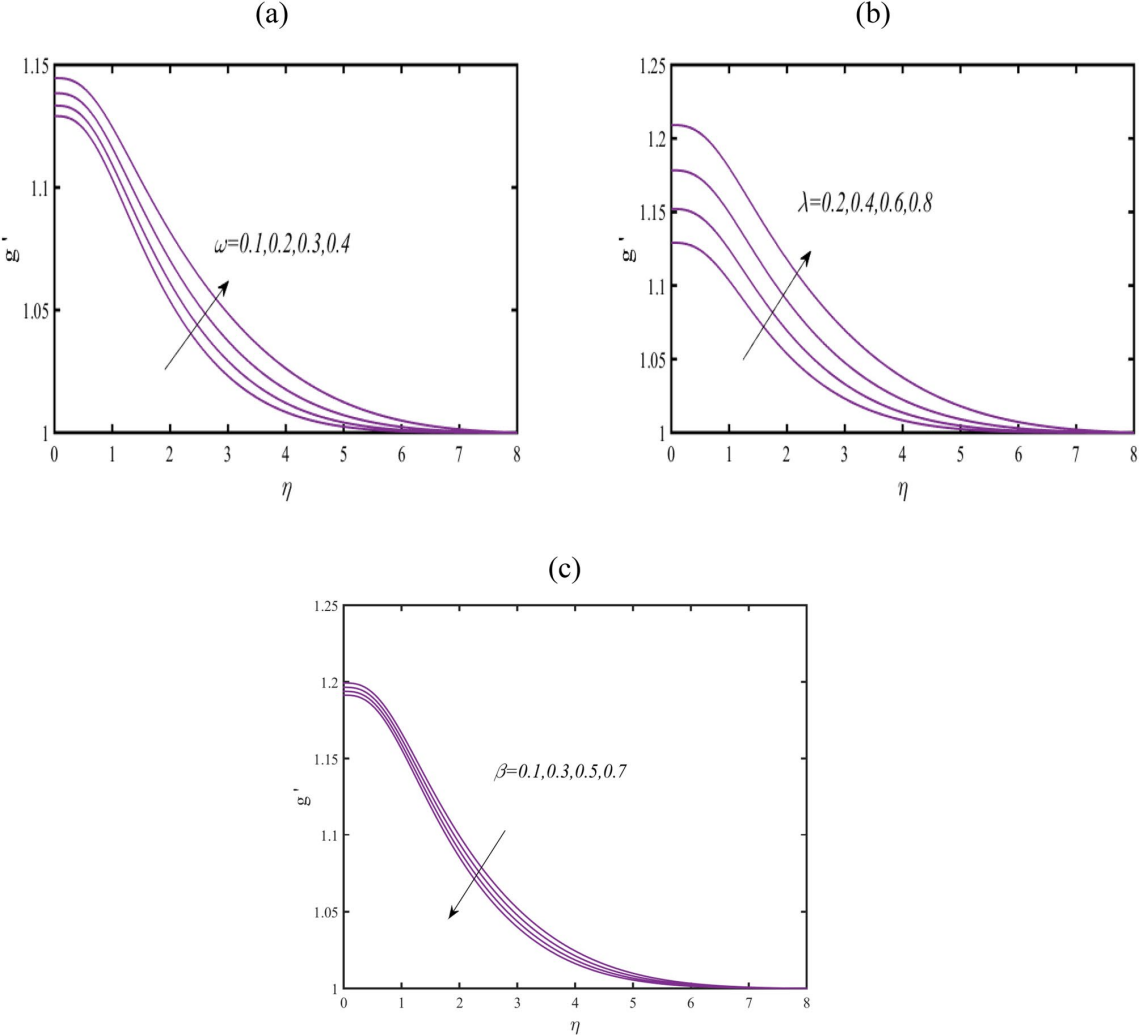
(**a**–**c**): Prediction of $g^{\prime}$ for (**a**) $\omega$ (**b**) $\lambda$ and (**c**) $\beta$.

Figure 4a reports the demonstration of induced magnetic field $g^{\prime}$ in view of reciprocal magnetic Prandtl constant $\omega .$ A strength impact of $\omega$ on $g^{\prime}$ is preserved. Such outcomes are associated to applications of induction of magnetic force. Figure 4b exploring the prediction in profile of $g^{\prime}$ against larger magnetic constant $\lambda .$ The increasing influence of $\lambda$ on $g^{\prime}$ is examined. Figure 4c suggesting the features of $\beta$ on $g^{\prime}$. The conveying results claims a depressing change in $g^{\prime}$ when $\beta$ get increases.

**Figure 5:**
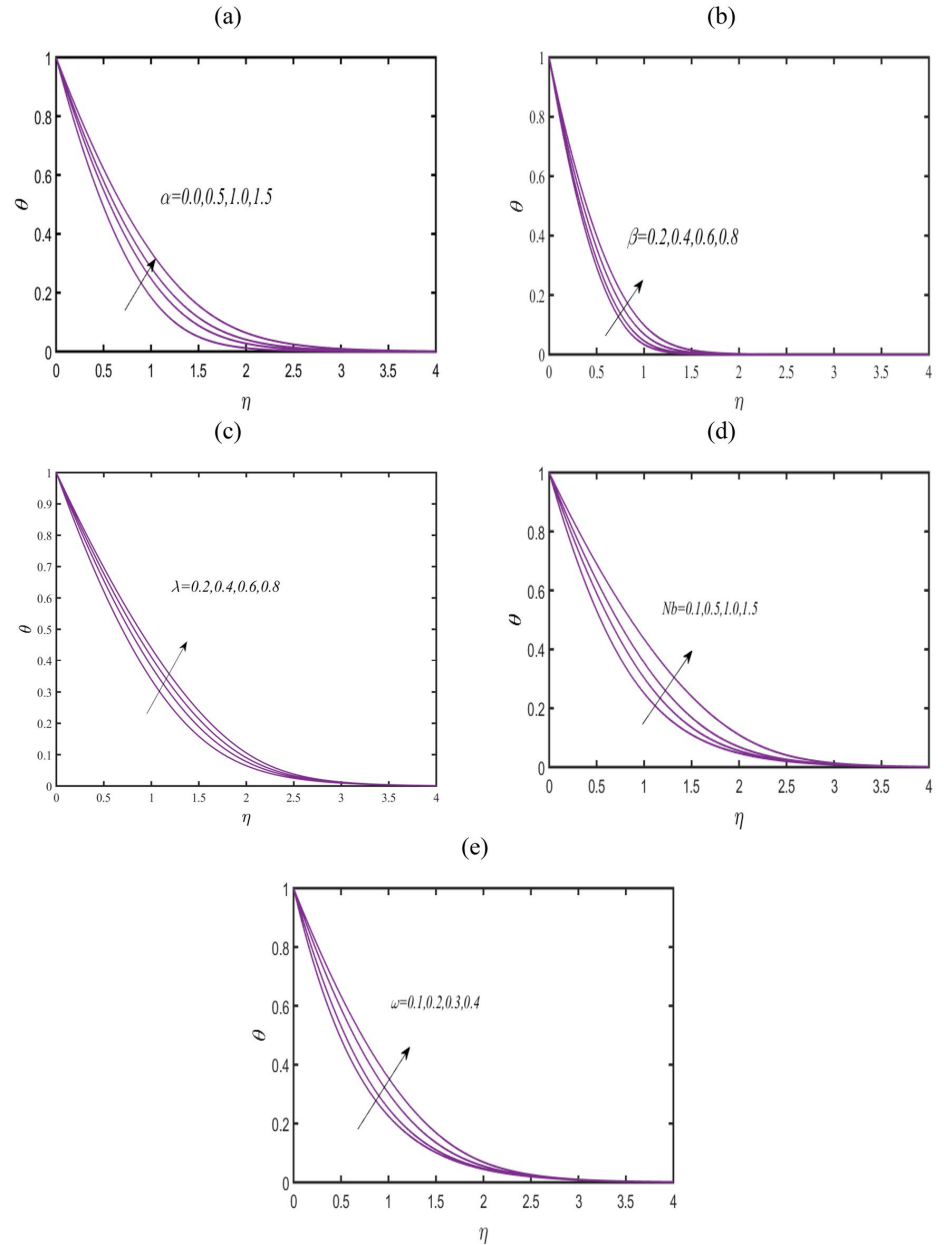
(**a**–**c**): Prediction of $\theta$ for (**a**) $\alpha$ (**b**) $\beta$, (**c**) $\lambda$, (**d**) $Nb$ (**e**) $\omega .$

Figure 5a announced the physical assessment of temperature field $\theta$ with variable thermal conductivity constant $\alpha .$ The utilization of variable thermal conductivity enhances the thermal phenomenon effectively. Based on such results, it is emphasized that transportation phenomenon can be effectively improves when fluid particles comprise variable thermal conductivity. Figure 5b presents the observations for $\theta$ by varying Casson fluid parameter $\beta$. The increment is deduced in $\theta$ due to privilege values of $\beta$. Physical insight behind this trend is due to distinct Casson rheology. Figure 5c reports that $\theta$ improves in progressive manner under the enhancement of magnetic constant $\lambda$. Figure 5d reports the insight of Brownian constant $Nb$ on $\theta .$ Boosted reflection of $Nb$ on $\theta$ have been incorporated. Such assessment is physical endorsed due to random movement of particles. During the random motion, a collision is noted between fluid particles which exclusively enhance the heat transfer rate. Figure 5e discussed the interesting features of reciprocal magnetic Prandtl constant $\omega$ on $\theta$. The enhancement in thermal phenomenon is obtained with $\omega$. The induction of magnetic force helps in improving the heat transfer prediction.

**Figure 6:**
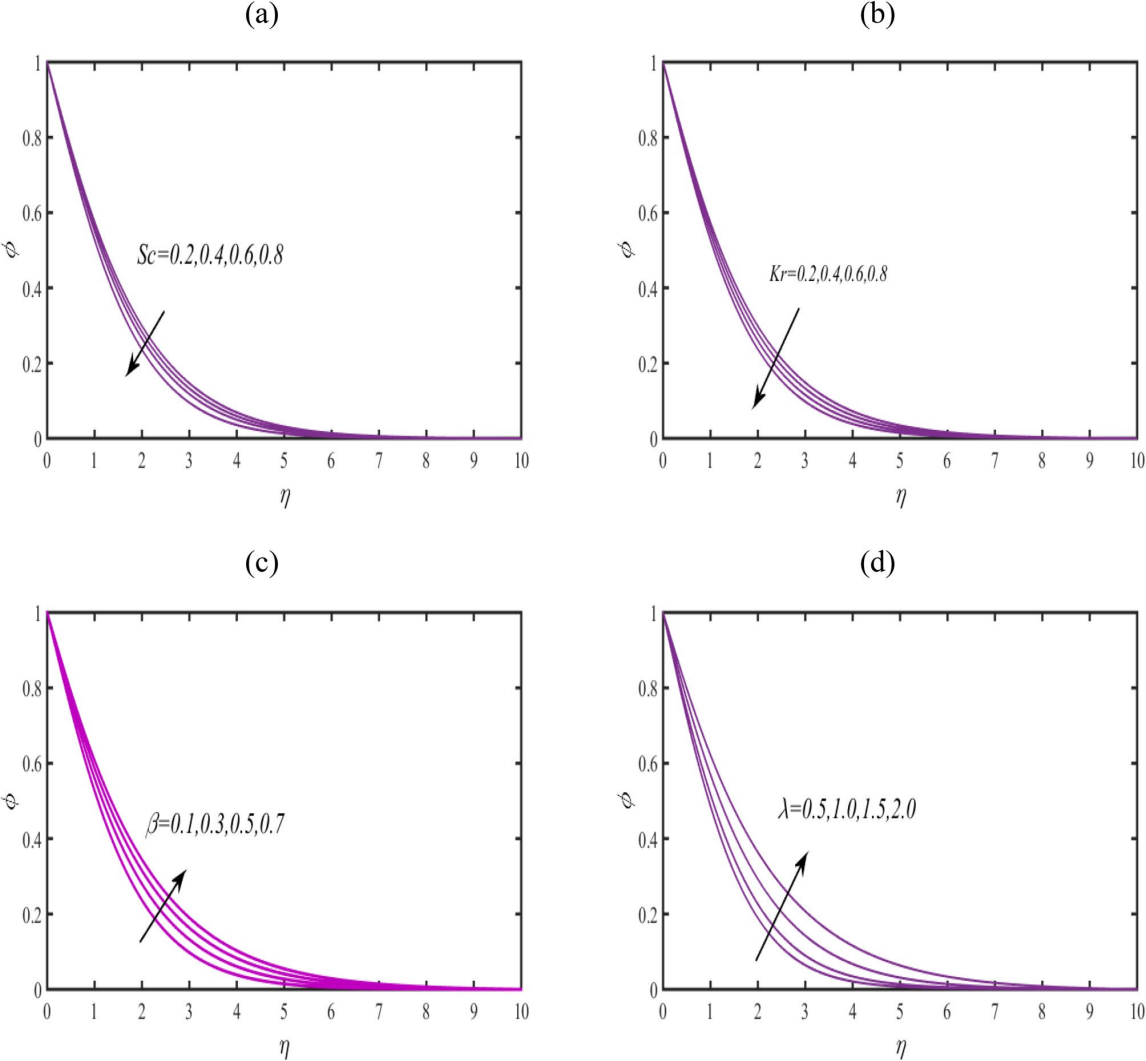
(**a**–**c**): Prediction of $\phi$ for (**a**) $Sc$ (**b**) $Kr$, (**c**) $\beta$ and (**d**) $\lambda$ (**e**) $\omega .$

Figure 6a announced the change in concentration field $\phi$ when Schmidt number $Sc$ get increasing impact. Slower change in $\phi$ is associated to increasing $Sc$. Physical exploration for such observations is lower mass diffusivity. Figure 6b predicted the objective of reaction constant $Kr$ for $\phi$. A control in chemical phenomenon is noticed when $Kr$ get improving values. Figure 5c claims that $\phi$ is increasing association with Casson parameter $\beta$. The concertation function gets increase for $\beta$. Figure 5d presented prediction in $\phi$ under the larger announcement of magnetic constant $\lambda .$ A boosted effected are announced in profile of $\phi$ under larger $\lambda .$

**Figure 7:**
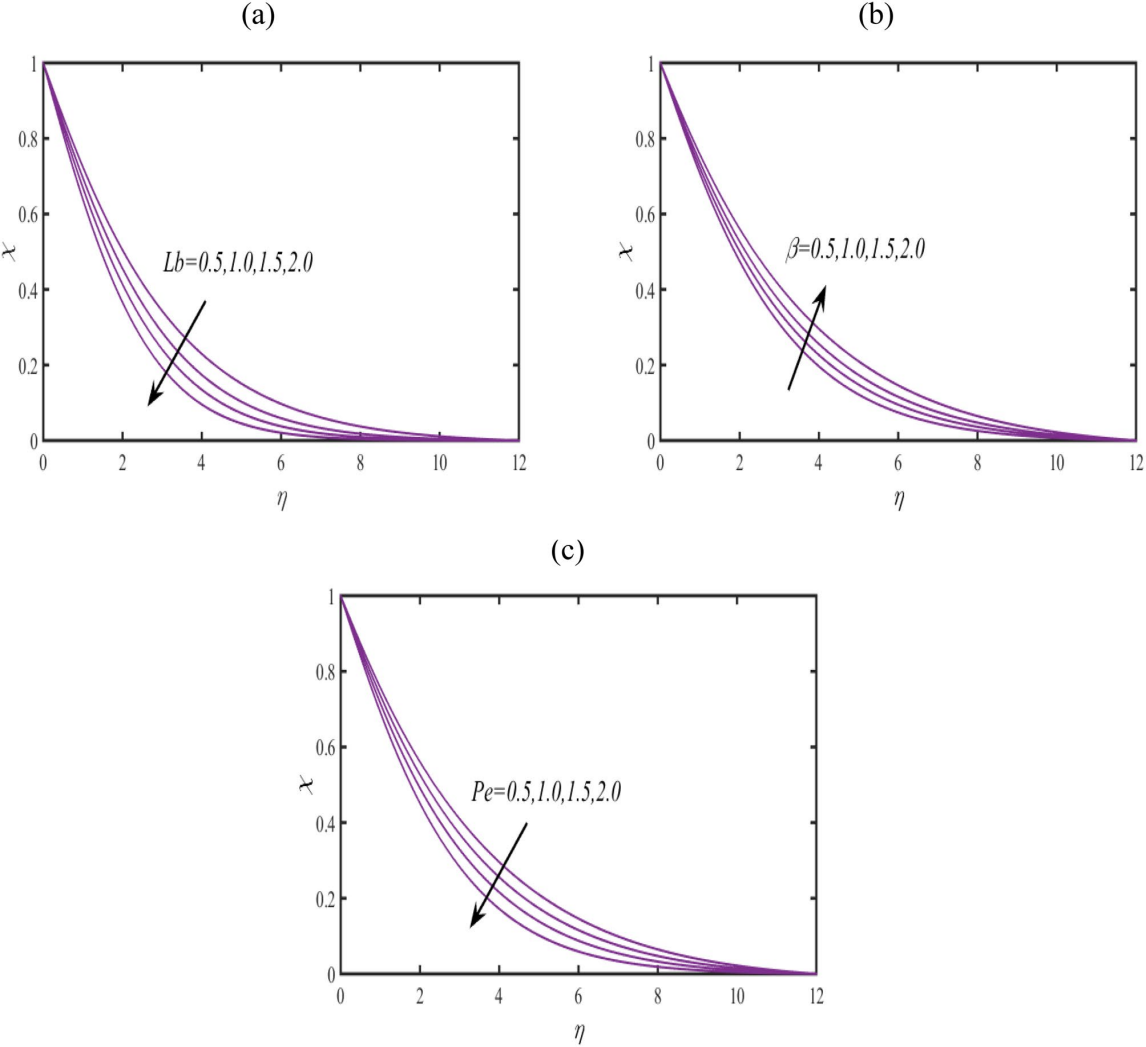
(**a**–**c**): Prediction of $\chi$ for (**a**) $Lb$ (**b**) $Pe$ and (**c**) $\beta$.

**Figure 8:**
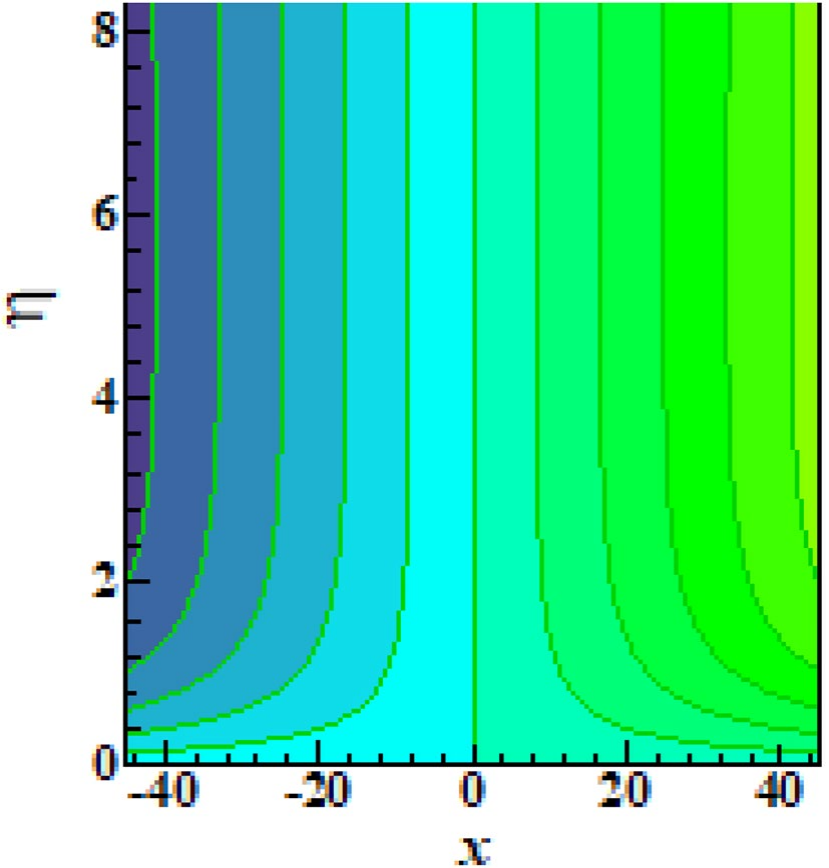
Illustration of streamlines.

Figure 7a examines the behavior of microorganism $\chi$ subject to larger bioconvective Lewis number $Lb$. The microorganism field $\chi$ reduces with $Lb$. The pattern of $\chi$ for Peclet number $Pe$ is explored in Fig. 7b. A contracted profile of $\chi$ is intended for $Pe$. Such declining phenomenon is due to smaller rate of motile diffusivity. Figure 7c reports that $\chi$ increases attentively for $\beta$. The stream lines are plotted in Fig. 8 to presents the flow behavior of moving fluid.

Numerical evaluation of $- f^{\prime\prime}\left( 0 \right)$, $- \theta^{\prime}\left( 0 \right)$, $- \phi^{\prime}\left( 0 \right)$ and $- \chi^{\prime}\left( 0 \right)$ by varying various parameters is presented in Table 2. The analysis is observation for $C$ and $\lambda$. A reduction in these quantities is noticed when values of $C$ varies between 0.1 to 0.8. However, the increasing observations are deduced when numerical values are chosen greater than 1. With increasing $\lambda$, $- f^{\prime\prime}\left( 0 \right)$, $- \theta^{\prime}\left( 0 \right)$, $- \phi^{\prime}\left( 0 \right)$ and $- \chi^{\prime}\left( 0 \right)$ declined.

**Table 2:** Numerical impact in $- f^{\prime\prime}\left( 0 \right)$, $- \theta^{\prime}\left( 0 \right)$, $- \phi^{\prime}\left( 0 \right)$ and $- \chi^{\prime}\left( 0 \right)$.

$C$	$\lambda$	$- f^{\prime\prime}\left( 0 \right)$	$- \theta^{\prime}\left( 0 \right)$	$- \phi^{\prime}\left( 0 \right)$	$- \chi^{\prime}\left( 0 \right)$
0.1	0.4	0.177589	0.38356	0.94426	1.156320
0.3		0.157563	0.375756	0.93265	1.276846
0.5		0.144512	0.363253	0.915676	1.314876
0.7		0.121513	0.346506	0.884262	1.353534
1.5		0.9413456	0.484556	1.020465	1.02345
2.0		0.993576	0.522422	1.122341	1.12624
2.5		1.25325	0.536788	1.263446	1.27538
	1.0	1.673257	0.633255	1.214775	1.22345
	2.0	1.615956	0.597335	0.995334	1.10465
	3.0	1.564667	0.556242	0.914385	0.933456
	4.0	1.532654	0.493533	0.875789	0.833243

## Major observations

The thermal inspiration for bioconvective transport of Casson fluid is exhibited with interaction of induced magnetic force. The analysis is proceeded under the variable thermal conductivity assumptions. Shooting simulations are performed for presenting the computational analysis. Major observations are:

- The increasing dynamic of velocity is preserved for magnetic constant and Casson fluid constant.
- The velocity profile enhanced for velocity ratio constant.
- The induced magnetic field increases for enhancing values of Reciprocal magnetic Prandtl constant.
- A reduction in induced magnetic field is examined for Casson fluid parameter.
- The heat transfer rate improves for induction of magnetic force associated with magnetic parameter.
- With improving Casson parameter and Reciprocal magnetic Prandtl constant, heat transfer boosted.
- The thermal process can be more effectively enriches when fluid thermal conductivity is variables.
- With increasing reaction constant, a reduction in concentration profile is exhibited.
- The concentration profile enriches for Casson parameter and magnetic constant.
- The microorganisms field boosted for Casson parameter.
- The current results provide directions to implementations and applications of induced magnetic force for hybrid nanofluid problems and performing the heat and mass transfer analysis for other non-Newtonian materaisl.

## Data Availability

The data that support the findings of this study are available from the corresponding author upon reasonable request.

## List of symbols

$$\left( {u,v} \right)$$: Velocity components $$\left( {{\text{ms}}^{ - 1} } \right)$$
$$T$$: Temperature $$\left( {\text{K}} \right)$$
$\left( {x,y} \right)$: Coordinates of plane $\left( {\text{m}} \right)$
$$\nu$$: Kinematic viscosity $$\left( {{\text{m}}^{2} \,{\text{s}}^{ - 1} } \right)$$
$$\mu$$: Dynamic viscosity $$\left( {{\text{Kg}}\left( {{\text{ms}}} \right)^{ - 1} } \right)$$
$\beta$: Casson fluid parameter
$$\rho_{f}$$: Density $$\left( {{\text{Kg}}\left( {\text{m}} \right)^{ - 3} } \right)$$
$$\sigma$$: Electrical conductivity $$\left( {{\text{S}}/{\text{m}}} \right)$$
$u_{\infty }$: Free stream velocity $\left( {{\text{Kg}}\left( {{\text{ms}}} \right)^{ - 1} } \right)$
$$D_{B}$$: Brownian coefficient $$\left( {{\text{m}}^{2} /{\text{s}}} \right)$$
$$D_{T}$$: Thermophoresis coefficient $$\left( {{\text{m}}^{2} /{\text{s}}} \right)$$
$K\left( T \right)$: Thermal conductivity as a function of temperature $\left( {{\text{W}}/{\text{m}}\,{\text{K}}} \right)$
$$\mu_{e}$$: Magnetic diffusivity
$H_{e}$: $x -$ Magnetic field at surface
$\tau_{f}$: Ratio amongst thermal capacity of nanoparticles to fluid
$k^{ * }$: Chemical reaction coefficient
$$b_{m}$$: Chemotaxis constant
$w_{m}$: Maximum cell swimming
$D_{m}$: Density of microorganisms
$\alpha$: Thermal conductivity coefficient
$\lambda$: Variables magnetic parameter
$$C$$: Velocity ratio
$\omega$: Reciprocal magnetic Prandtl number
${\text{Kr}}$: Chemical reaction constant
$${\text{Sc}}$$: Lewis number
${\text{Lb}}$: Bio-convective Lewis number
$${\text{Nb}}$$: Brownian constant
$${\text{Pe}}$$: Peclet number
$\sigma_{m}$: Motile difference constant
$${\text{Nt}}$$: Thermophoresis constant
$${\text{Nu}}$$: Nusselt number
$${\text{Sh}}$$: Sherwood number
${\text{Nn}}$: Motile density number
$${\text{Re}}$$: Reynolds number
